# Repeated Injection of Very Small Superparamagnetic Iron Oxide Particles (VSOPs) in Murine Atherosclerosis: A Safety Study

**DOI:** 10.3390/nano14090773

**Published:** 2024-04-28

**Authors:** Tobias Haase, Antje Ludwig, Anke Stach, Azadeh Mohtashamdolatshahi, Ralf Hauptmann, Lars Mundhenk, Harald Kratz, Susanne Metzkow, Avan Kader, Christian Freise, Susanne Mueller, Nicola Stolzenburg, Patricia Radon, Maik Liebl, Frank Wiekhorst, Bernd Hamm, Matthias Taupitz, Jörg Schnorr

**Affiliations:** 1 Charité—Universitätsmedizin Berlin, Corporate Member of Freie Universität Berlin and Humboldt-Universität zu Berlin, 10117 Berlin, Germany; antje.ludwig@dhzc-charite.de (A.L.); azadeh_dolatshahi@yahoo.com (A.M.); ralf.hauptmann@charite.de (R.H.); harald.kratz@charite.de (H.K.); susanne.metzkow@charite.de (S.M.); avan.kader@charite.de (A.K.); christian.freise@charite.de (C.F.); susanne.mueller1@charite.de (S.M.); nicola.stolzenburg@charite.de (N.S.); bernd.hamm@charite.de (B.H.); matthias.taupitz@charite.de (M.T.); joerg.schnorr@charite.de (J.S.); 2 Department of Radiology, Campus Mitte, Charitéplatz 1, 10117 Berlin, Germany; 3 Department of Cardiology, Angiology and Intensive Care Medicine, Deutsches Herzzentrum der Charité, Charitéplatz 1, 10117 Berlin, Germany; 4 DZHK (German Centre for Cardiovascular Research), Partner Site Berlin, 10117 Berlin, Germany; 5 Institute of Veterinary Pathology, Freie Universität Berlin, Robert-von-Ostertag-Str. 15, 14163 Berlin, Germany; lars.mundhenk@fu-berlin.de; 6 Department of Diagnostic and Interventional Radiology, Technical University of Munich, Ismaninger Str. 22, 81675 Munich, Germany; 7 Department of Experimental Neurology, Center for Stroke Research Berlin (CSB), Campus Mitte, Charitéplatz 1, 10117 Berlin, Germany; 8 Charité 3R|Replace, Reduce, Refine, Charité—Universitätsmedizin Berlin, Corporate Member of Freie Universität Berlin and Humboldt-Universität zu Berlin, 10117 Berlin, Germany; 9 NeuroCure Cluster of Excellence and Charité Core Facility 7T Experimental MRIs, Charité—Universitätsmedizin Berlin, 10117 Berlin, Germany; 10 Physikalisch-Technische Bundesanstalt, Abbestr. 2-12, 10587 Berlin, Germany; patricia.radon@ptb.de (P.R.); maik.liebl@ptb.de (M.L.); frank.wiekhorst@ptb.de (F.W.)

**Keywords:** iron nanoparticles, magnetic resonance angiography, hyperlipidemia, atherosclerosis

## Abstract

Citrate-coated electrostatically stabilized very small superparamagnetic iron oxide particles (VSOPs) have been successfully tested as magnetic resonance angiography (MRA) contrast agents and are promising tools for molecular imaging of atherosclerosis. Their repeated use in the background of pre-existing hyperlipidemia and atherosclerosis has not yet been studied. This study aimed to investigate the effect of multiple intravenous injections of VSOPs in atherosclerotic mice. Taurine-formulated VSOPs (VSOP-T) were repeatedly intravenously injected at 100 µmol Fe/kg in apolipoprotein E-deficient (ApoE KO) mice with diet-induced atherosclerosis. Angiographic imaging was carried out by in vivo MRI. Magnetic particle spectrometry was used to detect tissue VSOP content, and tissue iron content was quantified photometrically. Pathological changes in organs, atherosclerotic plaque development, and expression of hepatic iron-related proteins were evaluated. VSOP-T enabled the angiographic imaging of heart and blood vessels with a blood half-life of one hour. Repeated intravenous injection led to VSOP deposition and iron accumulation in the liver and spleen without affecting liver and spleen pathology, expression of hepatic iron metabolism proteins, serum lipids, or atherosclerotic lesion formation. Repeated injections of VSOP-T doses sufficient for MRA analyses had no significant effects on plaque burden, steatohepatitis, and iron homeostasis in atherosclerotic mice. These findings underscore the safety of VSOP-T and support its further development as a contrast agent and molecular imaging tool.

## 1. Introduction

Commonly used gadolinium-based contrast agents (GBCAs) are considered relatively safe for various medical applications and patient populations. However, there has been a rising concern regarding the accumulation of gadolinium in the brain and other tissues, potentially resulting in long-term adverse effects [1]. Particularly, patients with chronic kidney disease (CKD) are at risk of developing nephrogenic systemic fibrosis (NSF) and gadolinium retention [2]. These patients are also highly susceptible to atherosclerosis, where GBCA-enhanced magnetic resonance angiography (MRA) is routinely and repeatedly used for the assessment of vascular stenosis [3]. Given this concern, the exploration of alternatives to GBCAs for patients with renal dysfunction came back into focus.

Previously, our group developed a magnetic iron oxide nanoparticle (IONP)-based contrast agent featuring citrate as a stabilizing coating (VSOP-C184) for MRA applications [4]. These citrate-coated, very small superparamagnetic iron oxide particles (VSOPs) became a new class of particles characterized by a hydrodynamic diameter of approximately 6–8 nm. While showing excellent magnetic properties for MRI analyses, the size of a VSOP allows for long blood half-lives potentially without undergoing renal clearing, mitigating the risk of kidney accumulation in patients with renal dysfunction [5]. Beyond that, studies revealed an enhanced incorporation of VSOPs into atherosclerotic lesions [6], high-affinity interactions with altered glycocalyx of inflamed tissues, and a histological colocalization with glucosaminoglycans (GAGs) in the perivascular space [7]. Hence, VSOPs are not only a potential alternative to GBCAs for MRA in CKD patients but also hold promise for visualizing inflammatory changes of the extracellular matrix (ECM), potentially allowing for screening of early vascular changes or longitudinal monitoring of atherosclerosis progression.

Considering their biocompatible composition, citrate-coated iron oxide nanoparticles could generally be regarded as non-toxic. However, this assessment is primarily based on studies following a single intravenous or oral administration of nanoparticles [8,9]. In contrast, the clinical application of VSOPs potentially demands repetitive injections in a hyperlipidemic and atherosclerotic environment. Particularly in the context of pre-existing inflammatory diseases, concerns arise regarding organ toxicities resulting from iron overload-induced oxidative stress [10,11]. Moreover, the unresolved question of how elevated intra-plaque iron concentrations impact the progression of atherosclerosis and plaque stability further complicates the situation [12,13]. Any adverse effects of VSOPs on organ physiology and plaque progression could significantly limit their utility in MRA and plaque imaging.

Hence, the objective of this study was to explore the fate and potential pathological consequences of multiple doses of VSOPs in a hyperlipidemic setting of atherosclerosis-prone mice. To explore this, we employed our current taurine-formulated VSOP development candidate (VSOP-T).

## 2. Materials and Methods

### 2.1. Reagents and Nanoparticles

Chemicals and solvents were purchased from Sigma-Aldrich (St. Louis, MO, USA) unless otherwise stated. VSOPs were produced as previously described in [14] but with sodium hydroxide instead of ammonia. Nanoparticle size was measured by dynamic light scattering on a Zetasizer Nano ZS (Malvern Instruments Ltd., Worcestershire, UK) equipped with the Zetasizer Software Version 6.20. The samples were diluted with HEPES (Sigma-Aldrich, St. Louis, MO, USA) solution c(HEPES) = 10 mmol/L (pH = 7.4) to a final iron concentration of 1 mmol/L. Magnetic characterizations were performed at 40 °C and 40 MHz (0.94 T) using an MR spectrometer (Minispec mq 40; Bruker, Karlsruhe, Germany). Diluted nanoparticle solutions with iron concentrations between 0.1 and 1.5 mmol/L were prepared. Three solutions with different concentrations were measured for each sample. The relaxation coefficients R1 and R2 were obtained by linear fitting of T1 and T2 relaxation rates, and values were normalized to the iron concentrations. For the in vivo applications, taurine β = 10 g/L, mannitol β = 60 g/L, and meglumin β = 1 g/L were added to the particle suspension. The iron concentration was adjusted to 250 mmol/L and autoclaved. This formulation is hereinafter referred to as VSOP-T. Electron microscopy examinations were performed by the Zentraleinrichtung Elektronenmikroskopie (ZELMI) at the Technical University Berlin. High-resolution transmission electron microscopy (HRTEM) using a TECNAI G2 20 S-Twin (FEI-Company, Hillsboro, OR, USA) was used with accelerating voltage of 200 kV and a 300 mesh Cu-grid.

### 2.2. Ex Vivo MRI

Twenty-four hours after intravenous injection of 200 µmol Fe/kg VSOP-T in atherosclerotic mice (ApoE KO, 18 weeks on a Western-type diet, see material and methods section: animals and dosing regimen), animals were perfused with Karnovsky buffer (4% paraformaldehyde, 0.2% picric acid, 0.05% glutaraldehyde, and 0.1 mol/L sodium phosphate buffer). Mouse hearts with aortic arch and bifurcations were excised and embedded in low melting agarose and analyzed on 7 Tesla small animal MRI Scanner (7T BioSpec + 1H-Cryoprobe, Bruker, Ettlingen, Germany). For imaging of VSOP, T2*-weighted MRI was performed with a T2*_FLASH_3D sequence without fat suppression. The imaging parameters were repetition time (TR) = 31 ms, echo time (TE) = 8 ms, flip angle (FA) = 20°, bandwidth = 28,846 Hz, 1 average, coronal slice orientation with a field of view (FOV) of 19.2 × 19.2 × 19.2 mm, and a matrix dimension (MD) of 192 × 192 × 192, resulting in a scan time of 21:07 min).

### 2.3. In Vivo MRI

Images were acquired on a 3 Tesla MRI scanner (Magnetom Lumina, Siemens, Erlangen, Germany) using a solenoid (Tx/Rx) coil. The VSOP-T dispersion was diluted in mannitol (60 g/L) and intravenously injected through the tail vein in the anesthetized ApoE KO mouse at a dose of 100 µmol Fe/kg. The imaging was performed in coronal orientation with a 3D fast low-angle shot (FLASH) sequence before, right after, and in time intervals of 10 min post-VSOP administration. Further imaging parameters were repetition time (TR) = 8.4 ms, echo time (TE) = 3.83 ms, flip angle (FA) = 25°, field of view (FOV) = 118 × 58 mm, matrix size = 256 × 128 (reconstructed pixel size = 0.5 × 0.5 mm), and slice thickness = 0.4 mm, 96 slices, bandwidth of 454 Hz/Px, averages = 4, resulting in a scan time of 4:16 min, for the total experiment time of 55 min. Horos Open-Source Medical Image Viewer (version 3.3.6) was utilized for 3D maximum intensity visualizations. The signal-to-background noise ratio (SNR) was calculated by averaging values from three ROIs of equal size defined over the inferior vena cava at each time point. The post-contrast enhancement SNR was corrected by subtraction of pre-contrast enhancement SNR. The half-life was calculated as first-order exponential decay kinetics using GraphPad Prism software version 7 (GraphPad Software, San Diego, CA, USA).

### 2.4. Animals and Dosing Regimen

**Figure 1:**
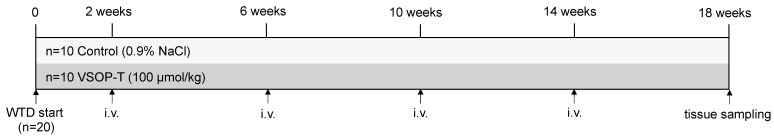
Dosing regimen and treatment schedule.

The animal experiments were approved and performed according to the local Guidelines and Provisions for Implementation of the Animal Welfare Act by the Charité-Universitätsmedizin Berlin, the regulations of the Federation of Laboratory Animal Science Associations (FELASA), and the local animal protection committee (LaGeSo, G000918). ApoE KO mice (B6.129P2-*Apoe^tm1Unc/J^*, JAX mice strain, Charles River) were kept under 12 h day/night cycle with water and food ad libitum. Atherosclerosis was induced by feeding 10-week-old male mice a Western-type diet (WTD, 21% fat, 19.5% casein, 0.15% cholesterol; Altromin) for 18 weeks. Mice were randomly divided into two groups (each *n* = 10), receiving four tail vein injections of 100 μmol Fe/kg VSOP-T or the same volume 0.9% NaCl (control) every four weeks starting two weeks after beginning of WTD (week 2, 6, 10, 14, Figure 1). Mice were sacrificed four weeks after the last VSOP-T injection (week 18) by blood withdrawal under isoflurane anesthesia (Forene, Abbot). Animals were transcardially perfused with PBS. Heart, aorta, spleen, liver, kidney, and lung were harvested and divided for snap freezing and formalin fixation.

### 2.5. Histology

Formalin-fixed specimens of liver and spleen were embedded in paraffin, and sections of 6 µm were cut and mounted on slides (SuperFrost plus, VWR International, Radnor, PA, USA). Tissue slices were stained with Prussian blue (1% potassium ferrocyanide in 1% HCl) and Hematoxylin and Eosin (HE). The histopathological analysis of stained hepatic and splenic tissue sections was carried out on scanned slides (Hamamatsu Nano Zoomer, Hamamatsu Photonics K.K., Hamamatsu, Japan). Grading of steatohepatitis was performed by a veterinary pathologist using a semiquantitative scoring system (scores 1–3) for macrovesicular steatosis, microvesicular steatosis, hypertrophy, and inflammatory foci. For histomorphometric analysis of atherosclerotic plaques, heart and aorta were carefully excised, fixed on a platform, and transferred to formalin. After formalin fixation for 48 h at 4 °C, heart base and aortic root with bifurcations were divided and embedded in paraffin. Sequential sections of 6 µm of the aortic root, aortic arch, and bifurcations were carried out and mounted on slides (see Figure A2). Sections were stained with Movat pentachrome staining (Morphisto, Offenbach am Main, Germany), scanned (Hamamatsu Nano Zoomer), and 3 sections of aortic root, aortic arch, and brachiocephalic trunk were analyzed using NDP view software (Version 2.7, Hamamatsu Photonics K.K., Hamamatsu, Japan).

### 2.6. Magnetic Particle Spectroscopy

The VSOP amount of the weighed tissue samples (weighed and filled into 0.2 mL sample tubes) was quantified by MPS. The measurements were performed employing a commercial MPS device (Bruker Biospin, Ettlingen, Germany) operating at a magnetic excitation field *B*_ex_ = 25 mT and a frequency *f*_0_ = 25 kHz. For quantification of the VSOP amount *m*(VSOP) in a sample, the measured third harmonic *A*_3_ of the MPS spectrum was normalized to the specific MPS amplitude A*_3,ref_ of a reference sample with known VSOP amount, where A*_3,ref_ = *A*_3,ref_/*m*(VSOP)_ref_ is the measured MPS amplitude *A*_3,ref_ normalized to the VSOP amount in the reference sample [15]. The VSOP concentration in a sample was obtained by normalizing *m*(VSOP) to the corresponding tissue mass (unit µg_VSOP_/g_tissue_). A detection limit *A*_3,min_ of the third harmonic was calculated according to the International Union of Pure and Applied Chemistry recommendation (IUPAC): *A*_3,min_ = mean(*A*_3,bgd_) + 3·SDV(*A*_3,bgd_), where mean(*A*_3,bgd_) and SDV(*A*_3,bgd_) represent mean and standard deviation of 100 background measurements without any sample. The ratio *A*_3,min_/A*_3,ref_ = 84 ng denotes the smallest amount of VSOPs that can be detected by MPS.

### 2.7. Tissue Iron Content

Between 10 and 40 mg of the frozen tissue were dried in a ceramic crucible at 100 °C for 2 h and then pyrolyzed at 500 °C in a muffle furnace for 4 h. The ash was dissolved in 400 μL hydrochloric acid c(HCl) = 6 mol/L and diluted to 5 mL with water. A total of 1 mL of this solution was mixed with 200 μL of the reducing and iron-chelating reagent [16]. The extinction of the sample was measured at 562 nm on a photometer against a blank (water). As standard, an iron solution with a concentration of 1 mg/L was used.

### 2.8. Serum Cholesterol and Triglyceride Measurement

Freshly collected whole blood samples were allowed to clot for 30 min at room temperature and centrifuged for 10 min at 2000× *g* at 4 °C. Supernatants were transferred to a new vial and snap frozen for further analyses. Cholesterol and triglyceride content of serum samples were analyzed using assay kits STA-384 and STA-396 (Cell Biolabs, San Diego, CA, USA) according to manufacturer’s protocols. Serum samples of C57Bl6/J mice on a standard diet served as a control.

### 2.9. Western Blot Analyses of Hepatic Proteins of Iron Storage and Transport

Snap-frozen liver samples (~50 mg) were homogenized with a small pestle in an Eppendorf tube in 400 µL RIPA lysis buffer consisting of 50 mmol/L Tris (pH 7.4), 1% Nonidet P-40, and 150 mmol/L NaCl supplemented with protease inhibitors (complete protease inhibitor, Roche Applied Science). Homogenates were incubated on ice for 10 min and subsequently centrifuged at 12,000× *g* for 15 min at 4 °C. The supernatants were transferred to a new tube, and protein concentrations were measured photometrically (Pierce BCA Protein Assay Kit, Life Technologies, Carlsbad, CA, USA) following the manufacturer’s instructions. Separation of total protein (10 μg per lane) was performed by gradient SDS-PAGE (4–12% Tris-Glycine gradient gel, Novex, Life Technologies). Separated proteins were transferred to PVDF membranes. Membranes were probed with the following antibodies: anti-ferritin (ab75973), anti-SLC40A1 (ab85370), and anti-transferrin (ab117310), all 1:1000 diluted from Abcam (Carlsbad, CA, USA). After incubation with the respective secondary antibodies conjugated with horseradish peroxidase, signals were detected with ECL plus (GE Healthcare Technologies, Chicago, Illinois, USA) using the chemiluminescence system Fusion Solo (Vilber Lourmat, Eberhardzell, Germany). Densitometric quantification of signals was performed using Image J (Version 1.52h). Amidoblack staining of the membrane served as control for equal protein loading and as standard for quantification.

### 2.10. Statistics

Data are presented as mean ± standard deviations. Diagrams showing histomorphometric data include individual data points. Densitometry data for Western blot are shown as median in box (from 25th to 75th percentile) and whiskers (from smallest to highest value) plot. Data sets were tested for outliers (ROUT method Q  =  1%) and normal distribution (Shapiro–Wilk test). In all comparisons with one independent variable, at least one group did not meet the criteria for normal distribution. Consequently, comparisons between two treatment groups were carried out by using the Mann–Whitney test, and comparisons of three groups (serum samples) were carried out by the Kruskal–Wallis test. The comparison of body weight development over time was carried out by two-way ANOVA with Sidak’s multiple comparison test. *p*-values of <0.05 were considered to be statistically significant. Statistics were calculated using GraphPad Prism 6 software.

## 3. Results

### 3.1. VSOP-T Characterization and Vascular Imaging

**Figure 2:**
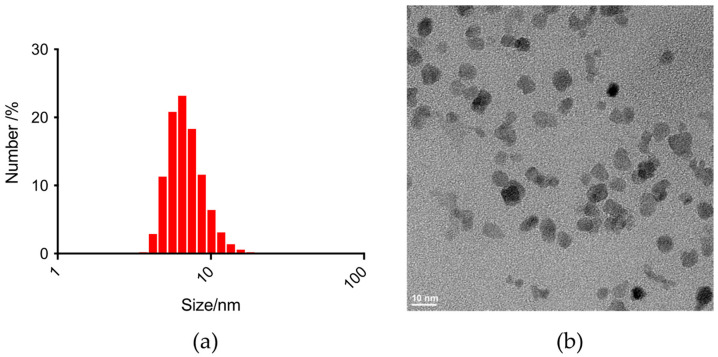
Size distribution is given by the number of particles of injected VSOP-T nanoparticles (**a**). Transmission electron microscopy (TEM) images show monocore structure and iron core sizes of approx. 6.5–7 nm for VSOPs (**b**).

Nanoparticles exhibited a hydrodynamic diameter of 7.1 ± 2.0 nm (Figure 2a,b) and demonstrated water relaxivities of r1 = 16.9 L·mmol*^−^*^1^·s*^−^*^1^ and r2 = 37.5 L·mmol*^−^*^1^·s*^−^*^1^. The formulation had an osmolality of 455 mOsm/L, and the pH was adjusted to 6.8–7.2 using meglumine, resulting in a final meglumine concentration of 0.212 g/L. The particles displayed a surface-bound citric acid content of 14.15 ± 0.047 g per 100 g of iron. Particle synthesis was conducted multiple times to establish reproducibility, and VSOP-T remained stable for several years under neutral pH conditions. Repeated size measurements revealed no evidence of aggregation or the presence of larger peaks, confirming the enduring stability and uniformity of the particles.

**Figure 3:**
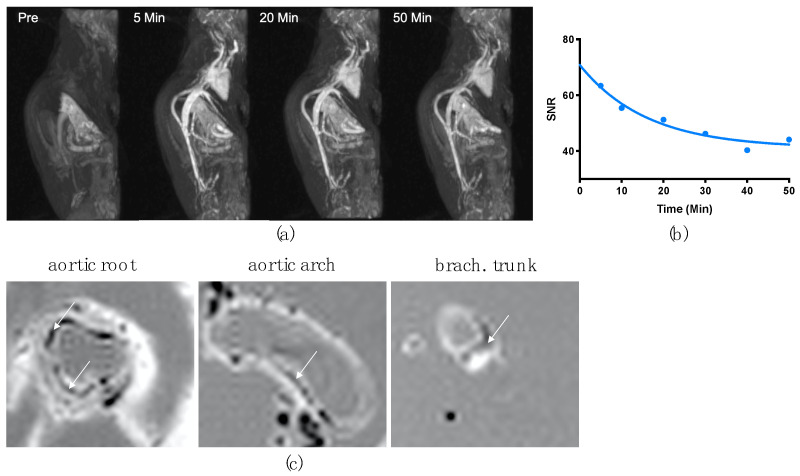
Vascular imaging with VSOP-T in the ApoE KO mouse. 3D maximum intensity projection (MIP) of angiography (T1-weighted images) with VSOP-T in a dose of 100 µmol Fe/kg, under same view angle pre-VSOP administration and at the given time points (**a**). Signal intensity ratio (SNR) development over time (**b**). Ex vivo MR images of aortae showing iron accumulations in regions of atherosclerotic plaques 24 h after injection (white arrows) (**c**).

Intravenous injection of 100 µmol Fe/kg VSOP-T in anesthetized mice enabled the angiographic imaging of heart and blood vessels with a blood half-life of one hour (Figure 3a,b). Immediately after intravenous injection, heart, aorta, and vena cava showed a strong positive contrast, proving that VSOPs are a feasible alternative for Gd-containing contrast media for MRI imaging. Consistent with our prior studies [6], iron accumulation in atherosclerotic vessel segments was confirmed using T2*-weighted ex vivo MRI scans 24 h post-injection. Intravenous application of VSOP-T caused a distinct signal loss within atherosclerotic plaques of the aortic root, aortic arch, and its branches (Figure 3c).

**Figure A1:**
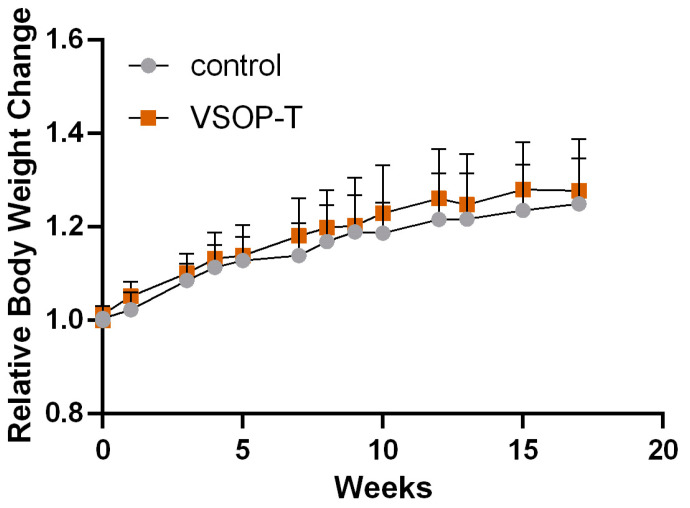
Relative body weight development of control and VSOP-T-treated mice over the period of 18 weeks on a Western-type diet. Means ± SD are shown (*n* = 10 animals per group).

Multiple intravenous VSOP-T injections at a dose of 100 µmol Fe/kg had no adverse effects on animal behavior and did not affect the normal weight gain of the mice (Figure A1). The macroscopic examination of organs at sacrifice revealed no differences in tissue structure or coloration between the analyzed groups.

### 3.2. Iron Distribution and Pathology

At animal sacrifice (four weeks after the last injection), VSOP-T was detected by MPS in liver and spleen in 6 of 10 and in the lung in 1 of 10 mice. VSOP-T was not detectable in the kidneys of VSOP-T-treated mice and in all analyzed organs of control mice (Table 1). These data were confirmed by photometric quantification of tissue iron contents, which revealed a significantly increased iron amount in liver and spleen of VSOP-T-treated mice compared to control mice and no increased tissue iron contents in lung and kidney (Table 1).

**Figure 4:**
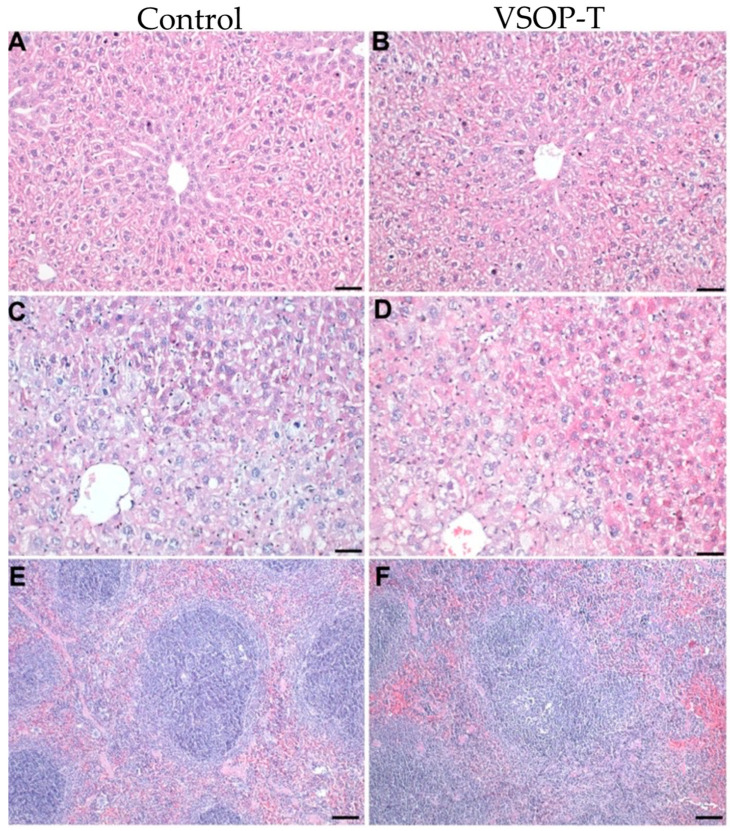
VSOP-T had no adverse hepatic or splenic effects. The characteristics of the hepatic lesions varied within the model; however, they were unaffected by the type of group. The hepatocytes of ApoE KO mice showed minimal (**A**,**B**) or severe vacuolization (**C**,**D**). In severely affected livers, the hepatocytes were variably sized, and the hypertrophied cells were predominately located centrolobular (**C**,**D**). The treatment with VSOP-T (**B**,**D**) had no effect on the hepatic phenotype. No differences were observed between the spleens of control (**E**) and VSOP-T-treated mice (**F**). Bars (**A**–**D**) = 50 µm. Bars (**E**,**F**) = 100 µm.

Histopathological analyses of liver and spleen revealed no differences between the control and VSOP-T-treated groups (Table 2). The livers of the ApoE KO mice were, in part, characterized by variable-sized hepatocytes with cytoplasmic vacuolation. The severity of the hepatic lesions varied within the model, as reported [17], but they were not affected by the administration of VSOP-T (Figure 4).

**Figure 5:**
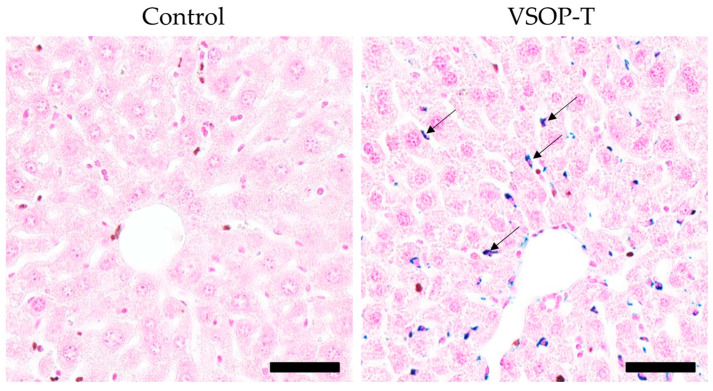
Visualization of iron in the hepatic sinusoidal lining cells. Compared to the liver of mice of the control group, the cytoplasm of hepatic sinusoidal lining cells of VSOP-T-treated mice stained intensively blue by Prussian blue staining (arrows). Bar = 50 µm.

Prussian blue iron staining revealed iron depositions in the liver of VSOP-T-treated mice compared to the control group. Liver sinusoidal lining cells, such as Kupffer cells, identified by the oval or comma-shaped nuclei and their association with the sinusoids, of VSOP-T-treated mice appear intensively blue by Prussian blue iron staining (Figure 5).

**Figure 6:**
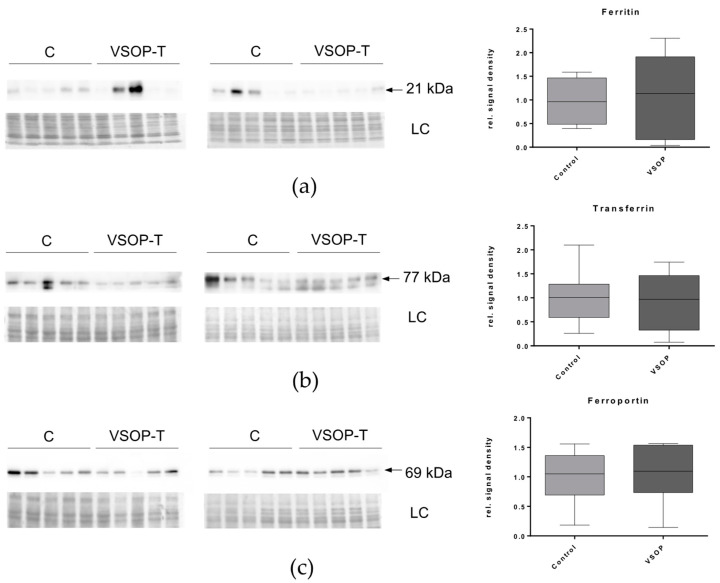
Analyses of hepatic expressions of proteins relevant for iron transport and recycling by Western blot: ferritin (**a**), transferrin (**b**), and ferroportin (**c**). Left panels show the Western blot results of 10 control mice (C) versus 10 VSOP-T mice (VSOP-T) separated on two membranes. Amido black staining served as loading control (LC). The right panels show the respective signal intensity analysis.

To quantitatively assess the expression of iron transport and recycling proteins, West-ern blot analysis in liver lysates were done. We observed no significant changes in the expression of ferritin, transferrin and ferroportin between control and VSOP-T treated groups (Figure 6). 

### 3.3. Impact on Atherosclerotic Plaques

**Figure 7:**
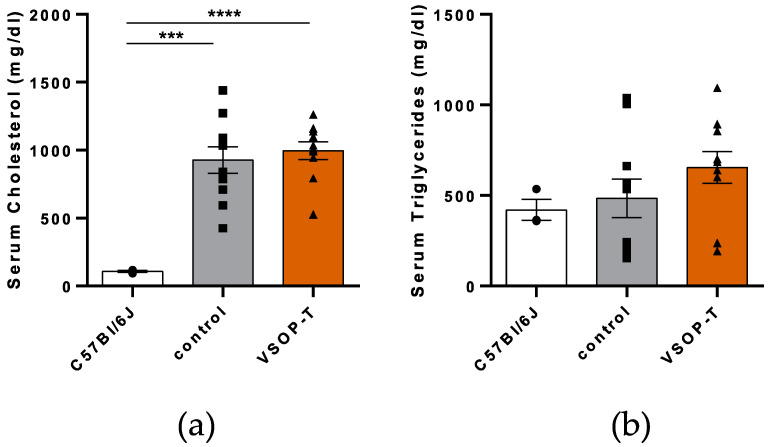
Serum cholesterol (**a**) and triglyceride levels (**b**) of control and VSOP-T-treated ApoE KO mice on HFD (*n* = 10 mice per group). For comparison, serum cholesterol and triglyceride levels of C57Bl6/J mice on normal diet (*n* = 3) were included in the diagrams. Data are shown as mean ± SD and individual data points. *** *p* < 0.001; **** *p* < 0.0001.

As expected, serum cholesterol was increased in ApoE KO mice on WTD compared to C57Bl6/J mice on a normal diet (Figure 7a). Within the ApoE KO group, no differences were detected between VSOP-T-treated and control mice (Figure 7a). The serum triglyceride levels were affected neither by strain/diet nor by VSOP-T treatment (Figure 7b).

**Figure 8:**
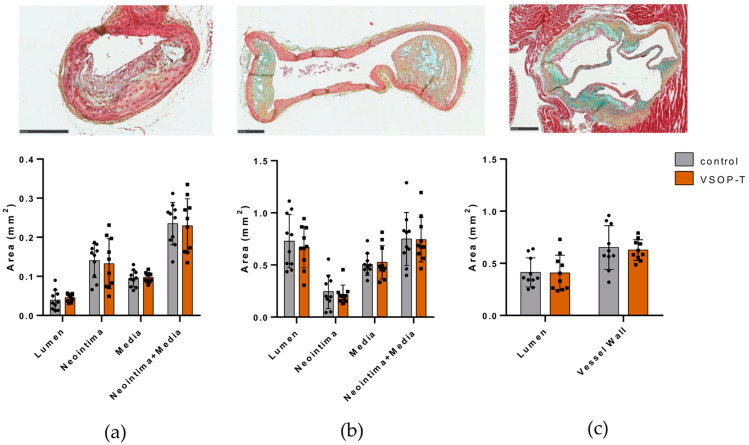
Movat pentachrome staining and histological evaluation of vascular segments from (**a**) brachiocephalic trunk, (**b**) aortic arch, and (**c**) aortic root of control (circles) and VSOP-T-treated mice (squares) (*n* = 10 mice per group). Data are shown as mean ± SD and individual data points. Bar = 250 µm.

**Figure A2:**
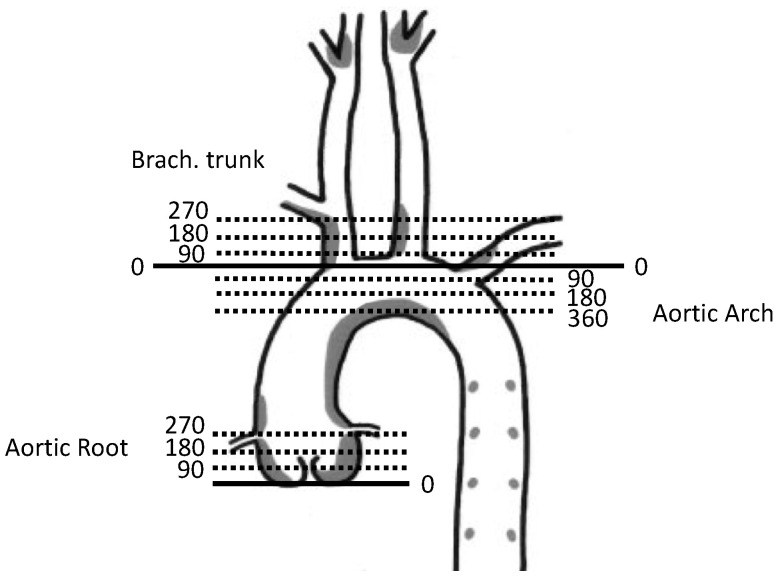
Tissue sections were used for histological analysis of atherosclerotic vascular changes in the three different vascular segments: brachiocephalic trunk, aortic arch, and aortic root. Consecutive sections were cut through the entire vascular segments, and sections at the indicated positions (dotted lines, µm) were stained, scanned, and analyzed. Image modified from [33].

Gross examination of the aortic arch with bifurcations showed atherosclerotic plaques to varying degrees in all mice. Analysis of three vascular sites susceptible to plaque formation, namely the brachiocephalic trunk, aortic arch, and aortic root, revealed characteristic atherosclerotic plaques with fatty/necrotic cores and medial thickening that significantly narrow the vessel lumen (Figure 8 and Figure A2).

We observed no statistically significant difference between control and VSOP-T-treated mice in terms of lumen narrowing, neointimal area, and medial area at all three atherosclerotic sites (Figure 8 and Table 3). Furthermore, there were no differences in maximal plaque thickness, lipid core area, and fibrous cap thickness between the treatment groups (Table 2).

## 4. Discussion

In the current study, we showed that multiple intravenous injections of VSOP-T in atherosclerotic mice had no adverse effects on the size and composition of atherosclerotic plaques. The multiple injections did not affect the general condition of the mice and had no adverse effect on liver status.

We used a novel VSOP-T formulated with taurine at a concentration of 10 g/L as an excipient for intravenous injection. Taurine is approved for intravenous applications in humans and is used for various therapeutic purposes [18]. Our repeated size measurements showed that the addition of taurine stabilizes the particles and prevents aggregation.

MRI imaging showed intravascular signal increases after intravenous application of 100 µmol Fe/kg with a blood half-life of one hour, allowing for long examination times and providing good visualization of vascular structures. Accumulation in atherosclerotic plaques enabled ex vivo MRI of atherosclerosis 24 h after VSOP-T injection. This confirms previous studies that showed that IONPs rapidly infiltrate atherosclerotic lesions in mice via transcytosis through the endothelium and finally accumulate in plaque resident macrophages [6,8,19,20,21].

It was previously demonstrated that intravenously injected VSOPs mainly accumulate in liver and spleen [8]. The current study confirmed elevated levels of VSOPs and related iron in liver and spleen due to an accumulation in sinusoidal lining cells, whereas hepatocytes appeared to not accumulate VSOP. The lack of VSOP-T-specific effects on the expression of hepatic proteins of iron metabolism could be attributed to the fact that the upregulation of iron metabolism proteins is confined to Kupffer cells and sinusoidal endothelial cells [8], which constitute a relatively small fraction of cells compared to the majority of hepatocytes. This suggests that the accumulation of VSOPs and related iron had no major impact on iron homeostasis in atherosclerotic mice. Hypercholesterolemia-induced steatohepatitis was not significantly altered by VSOP-T injection, indicating no VSOP-induced increase in oxidative stress or inflammation. Samples of kidney and lung showed no iron amounts above control levels. MPS measurements detected no VSOPs in the kidney. In one out of ten lung samples, we observed a positive VSOP signal by MPS; however, the non-elevated iron content measured by photometry pointed to a measurement error.

Of particular importance for the potential application of VSOP-T for cardiovascular disease imaging is our observation that the accumulation of iron-containing particles did not lead to any proatherogenic or plaque-destabilizing effects. Several studies have shown that atherosclerotic plaques are rich in endogenous iron, either in free form or bound to transport proteins [22,23,24], implicating a role in the pathogenesis of atherosclerosis [25,26]. Earlier investigations specifically designed to explore the effect of iron on cardiovascular diseases in animal models have yielded conflicting results [27,28,29,30,31]. The question of the role of iron in atherosclerosis was revisited in a recent study by Vichi et al. [32]. They showed that in ApoE KO mice carrying a mutation in the gene for ferroportin (FPN), which disrupts the hepcidin/FPN interaction, iron accumulation in the vessel wall exacerbated atherosclerosis. The observed pro-atherosclerotic effect in these mice was attributed to the occurrence of increased levels of non-transferrin-bound iron (NTBI) promoting lipid peroxidation and endothelial dysfunction. The observation that, in our experiment, the expression of hepatic proteins of iron metabolism in atherosclerotic ApoE KO mice remained unchanged even after multiple injections of VSOP-T indicated that iron homeostasis was not severely affected. We have previously observed that lysosomal degradation of VSOPs in primary mouse liver sinusoidal endothelial cells rapidly leads to increased levels of the iron storage protein ferritin. Ferritin also appears to be the “scavenger” of VSOP-related iron in vivo, as we observed by TEM that ferritin accumulated in close proximity to VSOP-filled endosomes in endothelial cells and macrophages of liver, spleen, and atherosclerotic plaques after a single VSOP injection in atherosclerotic LDLR^−/−^ mice [8]. We assume that the multiple injections of VSOP-T did not lead to harmful amounts of NTBI and consequently did not lead to an aggravation of atherosclerosis. We proved this by measuring the plaque load at three preselected atherosclerosis-prone sites, which showed no significant differences from the control group. The characteristics relevant to plaque stability, such as the size of the necrotic core and the thickness of the fibrous cap, also remained unaffected. This is in line with recent findings of Segers et al. using dextran-coated IONPs in a murine atherosclerosis model [12].

**Figure A3:**
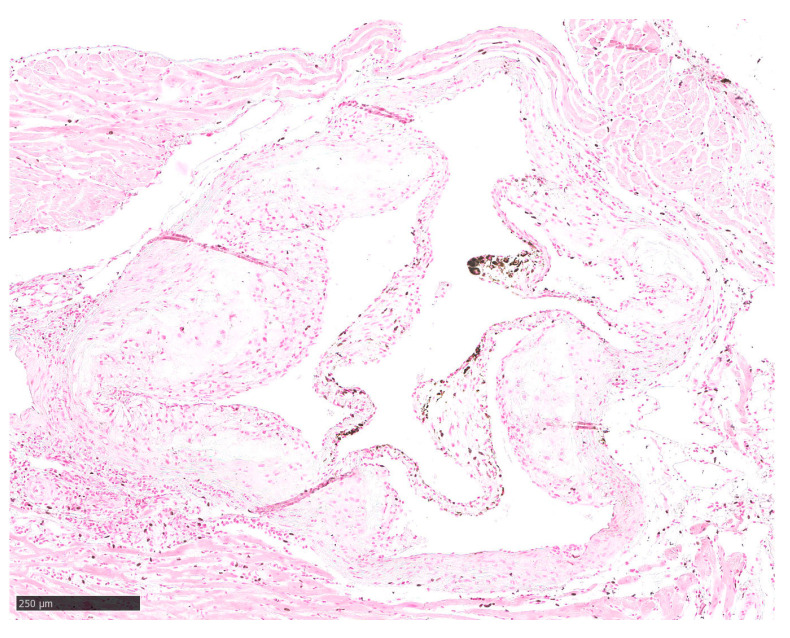
Prussian blue staining of the mouse aortic root, 4 weeks after the last VSOP-T treatment showing no iron staining. Bar = 250 µm.

In addition, iron staining of plaques and MPS measurements of thoracic aortae four weeks after the last VSOP-T injection revealed no detectable iron (Figure A3) or VSOP signal, demonstrating that VSOP-T use did not result in sustained pronounced iron accumulation in atherosclerotic plaques, although it was sufficient for ex vivo MRI imaging 24 h after injection.

### Limitations

The focus of this study was the assessment of the impact of multiple VSOP-T injections to evaluate their safety when used as a contrast agent in the background of atherosclerosis. Detailed data on molecular imaging and short-term biodistribution, as previously demonstrated for VSOPs [8], were not obtained. Longitudinal data on VSOP-T distribution and data for physiological and histological parameters for each VSOP-T treatment were not provided. Levels of NTBI were not measured. The conclusions drawn are limited to hyperlipidemia-induced atherosclerosis without additional complications such as impaired renal function or disorders of iron metabolism.

## 5. Conclusions

Multiple injections of the novel VSOP-T had no discernible impact on weight development, liver and spleen pathology, serum lipids, and atherosclerosis progression. As a result, we conclude that multiple VSOP-T doses, sufficient for magnetic resonance angiography (MRA), imaging of atherosclerosis, and other applications, are safe in an atherosclerotic environment. VSOP-T appears to be a promising candidate for further preclinical and clinical studies to be developed as a molecular imaging tool for the longitudinal monitoring of atherosclerosis progression.

## Figures and Tables

**Table 1:** Whole iron content (Fe mg_iron_/g_tissue_) measured by photometry and VSOP-T content (Fe MPS µg_VSOP_/g_tissue_) quantified by MPS of liver, spleen, lung, and kidney of control and VSOP-T-treated mice. Data are shown as mean ± SD (*n* = 10 mice per group). VSOP-T amounts below the MPS detection limit are given as n.d. * *p* < 0.05, ** *p* < 0.01 Mann–Whitney test, n.d. = not detected.

	Liver		Spleen		Lung		Kidney	
	Control	VSOP-T	Control	VSOP-T	Control	VSOP-T	Control	VSOP-T
Iron (mg/g)	0.10 ± 0.02	0.14 ± 0.03 *	0.47 ± 0.07	0.78 ± 0.24 **	0.032 ± 0.003	0.029 ± 0.002	0.041 ± 0.006	0.042 ± 0.004
VSOP (µg/g)	n.d.	18.28 ± 16.26	n.d.	23.25 ± 21.59	n.d.	0.72 ± 2.29	n.d.	n.d.

**Table 2:** Semiquantitative steatohepatitis score of HE-stained liver sections. Data are shown as mean ± SD (*n* = 10 mice per group).

Steatohepatitis Score		
	Control	VSOP-T
Macrovesicular Steatosis	0.20 ± 0.42	0.10 ± 0.32
Microvesicular Steatosis	1.40 ± 1.17	2.00 ± 0.82
Hypertrophy	1.70 ± 1.16	1.70 ± 0.82
Inflammatory foci	1.10 ± 1.37	1.20 ± 1.14

**Table 3:** Histological evaluation of vascular segments from brachiocephalic trunk, aortic arch, and aortic root of control and VSOP-T-treated ApoE KO-mice (*n* = 10 mice per group). Data are shown as mean ± SD.

	Brachiocephalic Trunk		Aortic Arch		Aortic Root	
	Control	VSOP-T	Control	VSOP-T	Control	VSOP-T
Lumen (mm^2^)	0.04 ± 0.03	0.05 ± 0.01	0.73 ± 0.26	0.67 ± 0.2	0.41 ± 0.14	0.41 ± 0.17
Neointima (mm^2^)	0.14 ± 0.04	0.13 ± 0.06	0.24 ± 0.16	0.22 ± 0.09		
Media (mm^2^)	0.09 ± 0.02	0.10 ± 0.01	0.51 ± 0.11	0.53 ± 0.16		
Neointima + Media (mm^2^)	0.24 ± 0.05	0.23 ± 0.07	0.75 ± 0.25	0.74 ± 0.21	0.65 ± 0.21	0.63 ± 0.1
Intima/Media Ratio	1.53 ± 0.56	1.36 ± 0.67	0.46 ± 0.24	0.44 ± 0.13		
Lumen Loss (%)	78.56 ± 13.75	70.65 ± 12.49	26.41 ± 13.47	25.71 ± 8.67		
Max. Neointima Thickness (mm)	0.24 ± 0.06	0.24 ± 0.09	0.20 ± 0.09	0.22 ± 0.05		
Lipid Core (mm^2^)	0.03 ± 0.02	0.03 ± 0.04	0.03 ± 0.03	0.03 ± 0.02	0.01 ± 0.01	0.01 ± 0.01
Min. Fibrous Cap	0.03 ± 0.02	0.02 ± 0.02	0.02 ± 0.01	0.02 ± 0.02	0.02 ± 0.02	0.014 ± 0.01

## Data Availability

Research data and supporting information are provided by the corresponding author, T.H.
